# The interface between clinicians and laboratory staff: A field study in northern Tanzania

**DOI:** 10.4102/ajlm.v3i1.126

**Published:** 2014-07-23

**Authors:** Coosje J. Tuijn, Elizabeth Msoka, Declare L. Mushi, Marion Sumari-de Boer, Jaffu Chilongola, Ankie van den Broek

**Affiliations:** 1 Royal Tropical Institute (KIT) Biomedical Research, Amsterdam, the Netherlands; 2 Kilimanjaro Clinical Research Institute (KCRI), Moshi, Tanzania; 3 Kilimanjaro Christian Medical University College, Tumaini University Makumira, Moshi, Tanzania; 4 Royal Tropical Institute (KIT) Health, Amsterdam, the Netherlands

## Abstract

**Background:**

Strengthening the communication and professional relationships between clinicians and laboratory workers is essential in order to positively change clinicians’ attitudes about the reliability of diagnostic tests, enhancing the use of laboratory diagnostics and, ultimately, improving patient care. We developed an analytical framework to gain insight into the factors that influence communication amongst health professionals.

**Objective:**

To explore whether the interaction between clinicians and laboratory workers influences the use of laboratory test results in clinical decision making.

**Methods:**

Four health facilities in northern Tanzania were selected using convenience sampling, whereas study participants were selected using purposive sampling. The quantitative and qualitative data collection methods included self-administered questionnaires; semi-structured, individual interviews; in-depth, individual interviews; and/or focus group discussions with clinicians and laboratory workers. Thematic content analyses were performed on qualitative data based on the framework. Descriptive statistical analyses of quantitative data were conducted using Microsoft Excel.

**Results:**

Contact between clinicians and laboratory professionals is seldom institutionalised and collaboration is rare. The clinicians believe collaboration with laboratory staff is a challenge because of the gap in education levels. Laboratory workers’ education levels are often lower than their positions require, leading to clinicians’ lack of respect for and confidence in laboratory professionals, which compromises the laboratory staff’s motivation.

**Conclusions:**

Hospital managers, clinicians and laboratory workers need to recognise the critical and complementary roles each professional plays and the importance of addressing the gap between them. Field application of the framework proved successful, justifying the expansion of this study to a larger geographical area to include additional healthcare institutions.

## Introduction

Medical laboratories play a significant role in the diagnosis, monitoring and treatment of diseases; yet the efficacy of the information they provide may be questioned because of several factors, including the capacity of the laboratory workforce, the laboratory infrastructure and the availability of equipment and materials, especially in low-income countries. Whilst improving the quality of laboratories is a solution, it does not always result in proper execution of tests.^1,2,3^ Other obstacles that must also be considered are the cultural beliefs of the patients, attrition of healthcare workers, physicians’ attitudes and inadequate supplies of consumables.^4^

Medical laboratory services offer essential information for diagnoses and/or treatment plans. The communication and interactions between laboratory and clinical health workers can influence physicians’ request behaviour and treatment interventions. Previous studies have shown that lack of communication is a barrier to effective healthcare.^5,6,7,8,9^ Improved communication between clinicians and laboratory workers is essential to changing clinicians’ attitudes about the reliability of diagnostic tests, possibly leading to increased use of laboratory diagnostics and, ultimately, improving patient care.^5^

This interface between clinicians and laboratory health workers is complex; the two groups may communicate face-to-face or by request and result forms, phone calls, text messages, e-mails or computerised forms. The factors that influence the mode of communication and shape the relationship between these two professional groups require further exploration. For this reason we constructed an analytical framework based on existing literature.^10,11,12,13,14,15^ After further literature searches, analysis of guidelines for laboratories and discussion with experts, a conceptual model was developed.^16^ The model addresses the phases where clinicians and laboratory workers interact; the organisational and personal factors affecting their interface; and the socio-political, economic and cultural environment within which the health facility operates. The objective of this study was to demonstrate and test the analytical framework and to gain insight into the relationship between clinicians and laboratory workers and into the factors that influence their interface, with the intention of later scaling up the study using a calculated sample size. The analytical framework includes three phases of communication (pre-analytical, analytical, post-analytical) during which clinicians and laboratory workers interact (Tables 1a and 1b).^15^ The testing process starts with a clinician ordering a test and sample collection, known as the pre-analytical phase. During the analytical phase, the sample is processed and analysed by laboratory staff. The post-analytical phase includes transfer of results from the laboratory back to the clinician. Each phase consists of organisational factors, subdivided into ‘identity’ and ‘management’, as well as personal factors, subdivided into ‘individual’ and ‘professional’. The primary aim of the study was to explore whether the interaction between clinicians and laboratory workers influences the use of laboratory test results in clinical decision making. By means of the framework quantitative and qualitative tools were designed. The results of this study provide information on the importance of the interface between clinicians and laboratory workers and may form a basis for larger studies in the future. The implications of our findings are useful for health institutions in any country.

**TABLE 1a:** Analytical framework. This framework was developed to test our conceptual model,^16^ and displays the organisational and personal factors playing a role during the three phases where clinicians and laboratory workers interact: pre-analytical, analytical and post-analytical.

Identity and management (Organisational factors)	Individual and professional factors (Personal factors)
**Management style:**	**The characteristics of the clinical and laboratory workforce of the organisation:**
Planning and implementation of regular meetings	Age
Monitoring and supervision systems	Level of education
Employment policies	CPD opportunities
Working environment	Years in service
-	Position in the organisation
-	Informal relationships between staffs

CPD, Continuing Professional Development.

**TABLE 1b:** Analytical framework. This framework was developed to test our conceptual model,^16^ and displays the three phases where clinicians and laboratory workers interact: pre-analytical, analytical and post-analytical. Each phase consists of organisational and personal factors (Table 1a).

Phase	General factors	Laboratory staff	Clinical staff
**Pre-analytical phase: The interface and the decision to use and perform a laboratory test correctly**	Availability of guideline/ SOP	Knowledge of availability and importance of tests	Knowledge of availability and importance of tests
	Availability of test request tools	Knowledge and attitude to ask for additional information from clinicians	Attitude towards the laboratory
	Under-/over-requesting of tests by clinicians	-	Providing the laboratory with sufficient patient information
	Competence/education of staff who are involved in the request, including the ‘intermediate health worker’	-	Patient wishes and needs
**Analytical phase: The interface during the (range of) tests**	Quality assurance mechanism in place	Asking for additional information	Taking time for (unplanned) discussion
	Time allocated to perform tests	-	Patient wishes and needs
	Human resources allocated to perform tests	-	-
	Availability and use of laboratory equipment and supplies and communication tools (phone, etc.)		-
**Post-analytical phase: The interface and the period between the test results and clinical decision making**	Availability of reporting forms	Appropriate and timely reporting	Knowledge of interpretation of test results
	Availability of reporting guidelines, including the role of the intermediate health worker	Understanding of how test results are used by clinicians	Trust in laboratory results
	Meetings on test results		Patient wishes and needs

## Research method and design

### Study design

This was an exploratory study, employing both quantitative and qualitative methods. Its purpose was to use tools to test the analytical framework and to better understand the factors that influence the interaction between clinical and laboratory workers. Most participants took part in a focus group discussion (FGD) immediately following completion of an anonymous, self-administered questionnaire (SAQ). If there were fewer than three participants for a FGD, in-depth, individual interviews were conducted. Semi-structured, individual interviews were used for hospital directors and heads of departments. FGDs and in-depth, individual interviews followed the same format and covered the same topics. The assessment of the data collection tools was done at a private, not-for-profit, faith-based, district hospital, where a group of clinicians and laboratory staff were invited to assess the tools.

### Study population

The study population included hospital directors, heads of clinical and laboratory departments, clinicians and laboratory staff. Staff came from three categories of health facilities: private, government; faith-based not-for-profit; and private for-profit. Four hospitals participated in the study: a non-government referral hospital with 450 beds; a private not-for-profit hospital with 150 beds; a government regional hospital with 300 beds; and a private for-profit health centre with 50 beds.

### Sample size, sampling procedures and data collection

Study sites were selected using convenience sampling, whereas study participants were selected using purposive sampling. As this was a pilot study, we did not determine a sample size. We contacted the sites and received verbal consent from the hospital directors for their participation. The interviewed staff members all signed written informed consent forms. Initially, a total of 48 staff members were asked to participate, including six clinicians and six laboratory workers from each of the four sites. However, at the time the study was conducted, only 35 staff members were present: 18 clinicians and 17 laboratory personnel. Amongst the latter were laboratory assistants and attendants who often perform the routine tests, namely, those who interacted most with clinicians (Tables 2a and 2b). SAQs were used to collect data from clinicians and laboratory staff at each hospital. After filling in the SAQs, staff members participated in either an FGD or an in-depth, individual interview, allowing for the opportunity to elaborate on the SAQs and further share their views. Interviews were carried out with hospital directors and heads of departments. All interviews and FGDs supplemented the SAQs and were conducted in Kiswahili for laboratory staff and in English for clinicians. Interviews were recorded via tape recorder or note taking. As outlined in the study protocol, FGDs involved six to 12 clinicians and three to nine laboratory staff members. These numbers were predetermined and agreed upon by the study team.

**TABLE 2a:** The demographic distribution related to the personal identities of the clinicians participating in this field study.

Demographic factors	Gender	
	Male (*N* = 11)	Female (*N* = 7)
**Ethnicity**		
Chagga	7	3
Pare	1	1
Masai	1	0
Makonde	0	1
Kalenjin	1	1
Ugandan/Maganda	1	0
Not provided	0	1
**Religion**		
Catholic	2	2
Lutheran	7	1
Muslim	0	1
Pentacostal	0	1
Seventh Day Adventist	1	0
Protestant	1	0
Not religious	0	1
Not provided	0	1
**Age**		
18–25	0	2
25–45	5	4
45–59	5	1
60+	1	0
**Personal qualifications**		
Diploma: Clinical Officer	5	3
Assistant Medical Officer	1	1
Mmed trainee	1	1
Medical Officer	3	1
Specialist	1	1
PhD or other postgraduate degree	0	0
**Position in the organisation**		
Head of Department	2	0
Principal Assistant Med Officer	0	1
Medical Officer	3	2
Senior Clinical Officer	5	1
Clinical Officer	1	2
Trainee	-	-
Not provided	-	1
**Institution**		
A	3	2
B	5	2
C	2	1
D	1	2
**Working experience**		
< 1 year	1	1
1–5 years	4	5
6–10 years	0	0
> 10 years	6	1
**Continuing professional development: Last time to attend a course**		
This year	4	3
1–3 years ago	1	1
4–6 years ago	1	1
7–10 years ago	1	0
> 10 years ago	4	1
Never	0	1
**Continuing professional development: Last time to attend a workshop/event**		
This year	5	4
1–3 years ago	2	2
4–6 years ago	0	1
7–10 years ago	2	0
> 10 years ago	1	0
Never	1	0

A, private not for profit; B, government regional; C, non government referral; D, private for profit.

**TABLE 2b:** The demographic distribution related to the personal identities of the laboratory workers participating in this study.

Demographic factors	Gender	
	Male (*N* = 8)	Female (*N* = 9)
**Ethnicity**		
Chagga	4	9
Sukuma	1	0
Kurya	1	0
Haya	1	0
Not provided	1	0
**Religion**		
Catholic	6	4
Lutheran	1	3
Muslim	0	1
Seventh Day Adventist	1	0
Not provided	0	1
**Age**		
18–25	1	0
25–45	3	7
45–59	4	1
60 +	0	1
**Personal qualifications**		
Laboratory attendant	1	6
Certificate laboratory assistant	4	2
Diploma laboratory technician	1	1
Laboratory technologist	0	0
Laboratory scientist	2	0
MSc in Microbiology	0	0
PhD or other postgraduate degree	0	0
**Position in the organisation**		
Laboratory director	2	4
Senior employee laboratory	3	3
Junior employee laboratory	1	2
Trainee	1	0
Not provided	1	0
**Institution**		
A	1	2
B	2	5
C	4	1
D	1	1
**Working experience**		
< 1 year	2	0
1–5 years	2	2
6–10 years	1	1
> 10 years	3	6
**Continuing professional development: Last time to attend a course**		
This year	3	0
1–3 years ago	2	2
4–6 years ago	1	1
7–10 years ago	1	0
> 10 years ago	1	5
Not provided	0	1
**Continuing professional development: Last time to attend a workshop and/or event**		
This year	4	3
1–3 years ago	3	1
4–6 years ago	1	1
7–10 years ago	0	0
> 10 years ago	0	2
Not provided	0	2

A, private not for profit; B, government regional; C, non government referral; D, private for profit.

### Data analysis

Data were collected within a period of five working days at the end of November 2011 and analysis was carried out throughout 2012. The individual interviews and FGDs were transcribed, translated and analysed by a social scientist and a research nurse. Qualitative data were analysed independently and manually, using a thematic framework approach involving data familiarisation, coding and development and categorisation of themes. Coding of collected qualitative data was driven by the developed framework (Tables 1a and 1b), whereby common words were sorted together, following an inductive method of code-creation. Once a theme was identified and reviewed, categorisation and corresponding codes were developed to sort and organise the data. After reading through the data, the two independent researchers discussed the codes and themes until they agreed on each one. Quotations were used to support and clarify the information provided using an editing analysis style. Descriptive statistical analysis of quantitative data from the structured questionnaires were carried out using Microsoft Excel.

## Ethical considerations

Ethical clearance was obtained from the Kilimanjaro Christian Medical University College Research and the Ethical Review Committee of Tumaini University, Makumira (Research Ethical Clearance certificate number 448 from Research Proposal 467). Verbal and written consent for the SAQs, interviews and FGDs was provided by study participants. Confidentiality was assured at all stages of this study, through the use of coding for sites and participants. No names or data can be traced back to individual participants.

## Trustworthiness

The results of this study are based on actual findings as described in the research method and design section. Qualitative research (FGDs and in-depth interviews) were complementary to results obtained from the SAQ. The experimental design of this exploratory study is reliable and valid and the procedures of qualitative and quantitative research used in this study are according to standardised methods, as laid out by Varkevisser, Pathmanathan and Brownlee.^17^

## Results

### Participants

Pre-testing of the data collection tools, firstly, through discussions with clinical and laboratory staff at a referral hospital and secondly, by group discussions at a district hospital, allowed researchers to strengthen and modify the data collection methods. In total, 35 questionnaires were administered to 18 clinicians and 17 laboratory staff members, an estimated one-third of the official staff number, according to the heads of departments.

### Factors influencing the interface

#### Organisational factors

**FIGURE 1:**
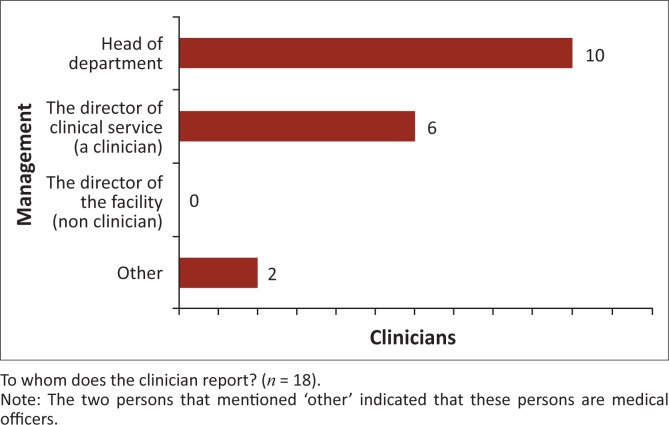
Factors related to the management of the organisation, response of clinicians.

**FIGURE 2:**
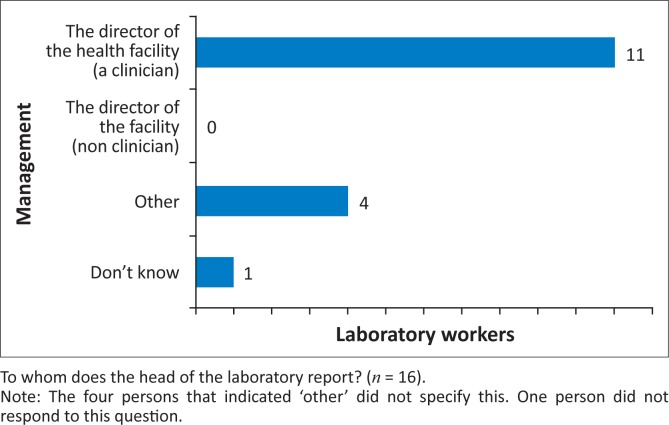
Factors related to the management of the organisation, response of laboratory staff.

**Management factors:** The analysis of the SAQs showed that 11 of the 18 clinicians (61.1%) and eight of the 17 laboratory staff (47.1%) were aware of the availability of rules and guidelines for requesting tests and reporting results. Of those remaining, one clinician (5.5%) and nine laboratory staff (52.9%) said there were no guidelines, whilst six clinicians (33.3%) did not know whether or not guidelines existed. These findings were also evident in the FGDs. Those who were aware that there are guidelines in place noted that there is little time to adhere to them because of staffing shortages and an insufficient supply of reagents. Other factors that the study group cited as impacting on communication in relation to management of the organisation included the clinicians’ doubts about laboratory test results and uncertainty as to whether Standard Operational Procedures are followed as well as the awareness of the persons to whom clinicians and laboratory staff report (Figure 1 and Figure 2). Furthermore, the lack of competent and highly-educated laboratory staff was cited by clinicians as being a barrier to effective communication; in many health facilities, only laboratory attendants and assistants are present to perform tests and they sometimes lack the communication skills of a more highly-educated laboratory technician. In FGDs with laboratory workers, it was noted that clinicians do not always use the test results with which they are provided. Patients sometimes ask clinicians to prescribe treatment straight away as the waiting time to be tested in the laboratory can be very long. Clinicians sometimes agree to this request in order to save time. Laboratory staff also mentioned that nurses and sometimes patients play a role in the contact between the clinicians and laboratory staff. In some health facilities, nurses collect the sample request from the wards and are responsible for transfer of results from the laboratory back to the clinician. This supports the findings of the quantitative data analysis.

**Identity:** Ten (55.5%) of the 18 clinicians and nine (52.9%) of the 17 laboratory workers involved in the survey worked in a referral hospital. Others worked in a faith-based, public or private, for-profit facility or in a general laboratory or health facility.

### Personal factors

Within the personal factors, individual and professional subfactors (qualitative data: quotes) were supportive of and in agreement with the quantitative data.

**Individual factors:** The personal factors of the clinicians (Table 2a) and laboratory staff (Table 2b) investigated in this study were ethnicity, religion, age and professional qualifications. Data are displayed by gender, position in the organisation and the last time a course or workshop was attended.

**Professional factors:** Laboratory staff members indicated that clinicians regularly devalue their services. Whilst seven of the 17 laboratory staff members (41.1%) believed that clinicians understand what laboratory workers do and six (35.3%) believed that clinicians know how to interpret the results when making clinical decisions, three (17.6%) noted that clinicians do not wait for laboratory test results before starting treatment and one (5.8%) noted that test ordering is not always specific. Furthermore, in the FGDs, nearly all of the laboratory staff members from public and faith-based organisations expressed that they lack recognition from clinicians, a sentiment also expressed by staff from the private health facility as being a contributing factor for their lack of motivation.

**FIGURE 3:**
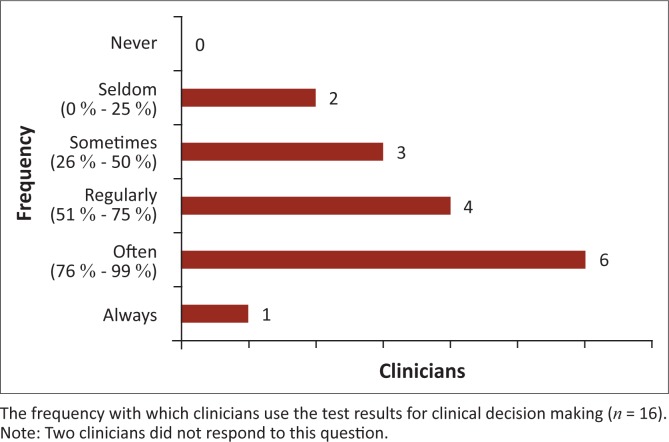
Professional factors. The perceptions of clinicians regarding the frequency with which test results are used for making clinical decisions.

**FIGURE 4:**
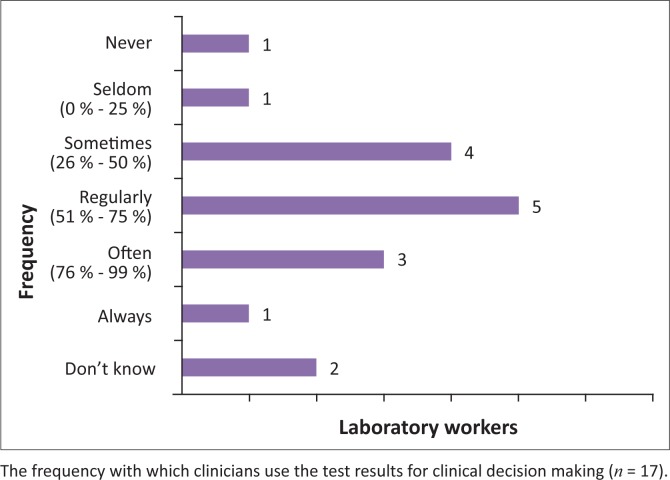
Professional factors. The perceptions of laboratory workers regarding the frequency with which test results are used for making clinical decisions.

Further playing a part in the complicated relationship between clinicians and laboratory staff is the perceived frequency of use of test results for clinical decision making. Only six clinicians (33.3%) and three laboratory workers (17.6%) claimed that test results are often used in clinical decision making. One laboratory staff member (5.8%) believed that clinicians never use the test results (Figure 3 and Figure 4).

Six of the 18 clinicians (33.3%) do not always trust the laboratory results; 10 (55.5%) mentioned that the waiting time for test results is too long; and eight (44.4%) were not satisfied with the type of tests that can be performed and also believed that the reporting of test results is not done properly. Four clinicians (22.2%) believed that the quality of laboratory services was weak or substandard. Several times during the FGDs, laboratory workers noted that lack of equipment contributes to poor quality output. Laboratory staff in the private hospitals pointed out the issue of lack of reagents and mentioned that expired reagents may be in use, further compromising clinicians’ confidence in the laboratory test results.

**FIGURE 5:**
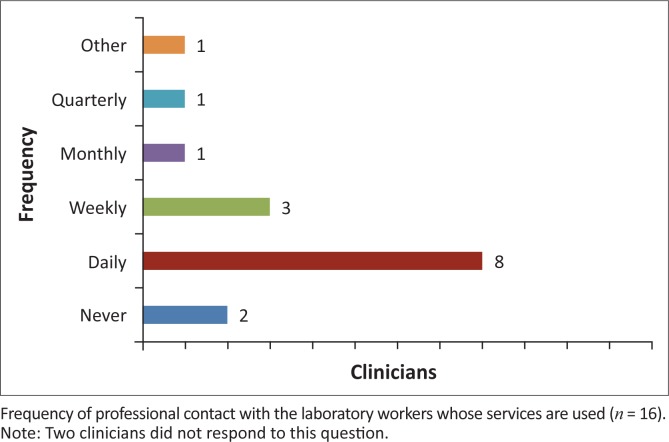
The frequency of professional contact between clinicians and laboratory workers. Eight of 16 clinicians reported having daily interactions with laboratory workers.

**FIGURE 6:**
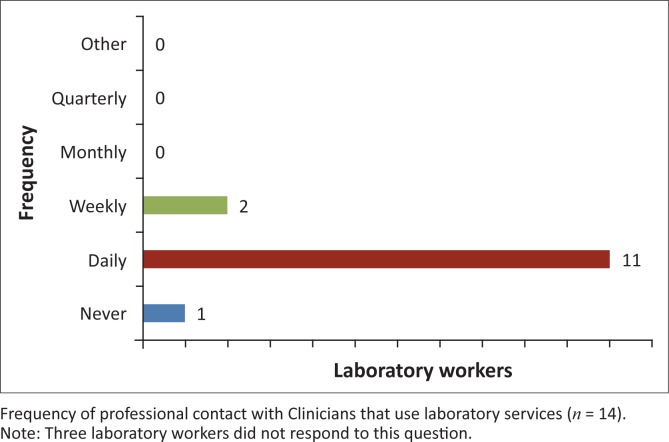
The frequency of professional contact between clinicians and laboratory workers. Eleven of 14 laboratory workers reported having daily professional contact with clinicians.

In spite of the complicated relationship between clinicians and laboratory workers, a majority in both groups must interact on a daily basis (Figure 5 and Figure 6). Yet, when grievances arise, the two groups use different avenues to address them. When laboratory staff members have problems with clinicians, they often discuss them within their own professional group; however, when a clinician has a complaint, he or she will often approach the individual laboratory worker or laboratory manager. Whilst issues like these may be discussed broadly in staff meetings at most hospitals, the majority of those surveyed at the private not-for-profit hospital pointed out that the department was so small that organising meetings to discuss problems seemed unnecessary.

Poor reporting was identified as being a factor that contributed to inadequate communication between clinicians and laboratory staff. In some cases, either the handwriting was misinterpreted, or test requests or test results were incomplete. In addition, it was noted that communication only occurs between the groups when the need arises. Whilst most staff members noted that communication and positive interactions between laboratory workers and clinicians are crucial, there is no managerial support, formalised system or motivation to maintain regular meetings or contact between clinicians and laboratory staff.

## Discussion

The main objectives of this study were to test the analytical framework; to gain a better understanding of the factors that influence the interface between clinicians and laboratory health workers; and to investigate the impact of the use of laboratory test results on the way clinicians and laboratory workers interact to deliver effective and improved healthcare. Our research results show that the roles of laboratory workers within the organisation are not determined by education levels, but by availability. According to clinicians, differences in levels of education lead to a lack of trust between clinicians and laboratory staff, impacting negatively on their collaboration and communication and creating a climate of distrust.

Poor communication between clinicians and laboratory staff further causes hostility when clinicians request a large number of tests, unaware of the high workload of the frequently understaffed laboratory. In all three phases of communication where clinicians and laboratory workers interact (pre-analytical phase: ordering of tests, sample collection; analytical phase: sample processing and analysis; post-analytical phase: results transfer), clinicians discuss their complaints and grievances with the laboratory staff more often than vice versa, suggesting that hierarchy plays a role in the dynamic between the two groups. Despite their different perceptions of a variety of issues, the groups agreed that clinicians were sometimes reluctant to use test results for clinical decision making. All in all, the issue of ineffective communication between clinicians and laboratory staff on patient care and worker dissatisfaction remains largely unresolved, providing a major source of frustration for staff and resulting in inefficiency in expected outputs.^5,6,7^ This study has increased the understanding of the interface between clinicians and laboratory workers and highlighted its importance in improving the quality of patient care. Scaling up data collection in a larger group of health facilities is essential with regard to quantifying our findings. This will enable hospital managers to make suggestions for improvements, such as refresher training courses that cover communication skills, as well as involving clinical and laboratory staff, nurses and patients. The research may also motivate clinicians and laboratory managers to pay more attention to the International Organization for Standardization (ISO)^18^ stipulations regarding communication. It is hoped that the findings from this study and similar future studies will, ultimately, improve the quality of patient care and communication between clinical and laboratory staff.^19^

## Limitations of this study

Clinicians were sometimes rushed during the interview, as patients were waiting for assistance. Only those staff who were on duty participated at the time of this study, which may have biased our results toward the perspectives of laboratory staff with lower education levels, since many more highly-educated staff were out in the field, in training, on holiday or had resigned. The perspectives of nurses and patients were not included in this study. Laboratory staff often had difficulties in filling in the English SAQ. Some questions were not clear and allowed multiple answers, which were adjusted for in the analysis. There were inconsistencies between the questionnaires for clinicians and those for laboratory workers. The outcome of our study was based mainly on qualitative data; the quantitative components were limited. In addition, the sample size of this study was too small to draw firm conclusions. As this was a pilot study, mainly descriptive statistical methods were used. This may limit the interpretation of the data presented, but it does provide valuable information on the interface between clinicians and laboratory workers and on the effectiveness of the framework to assess this interface. These insights may be used for future studies.

## Conclusion

By combining quantitative and qualitative information, some insight emerged regarding the relationship between clinicians and laboratory workers and the perspectives that contribute to their sometimes problematic interactions. This explorative study has given us additional information on factors that influence the interface between clinicians and laboratory workers and shown the effectiveness of the analytical framework. The findings and discussions also provided information for the improvement of our analytical framework, indicating the need for inclusion of nurses and patients in future studies. The findings from this study underscore the relevance of the subject: the daily struggle of hospital managers, clinicians and laboratory workers to recognise the critical role each plays in providing efficient and reliable healthcare. Performing this field study has provided information on the complexities of the interface between clinicians and laboratory staff and its impact on clinician decision making. The results justify expanding this study to a larger geographical area to include more health institutions.
